# Dislocation Filter Based on LT-GaAs Layers for Monolithic GaAs/Si Integration

**DOI:** 10.3390/nano12244449

**Published:** 2022-12-14

**Authors:** Mikhail O. Petrushkov, Demid S. Abramkin, Eugeny A. Emelyanov, Mikhail A. Putyato, Oleg S. Komkov, Dmitrii D. Firsov, Andrey V. Vasev, Mikhail Yu. Yesin, Askhat K. Bakarov, Ivan D. Loshkarev, Anton K. Gutakovskii, Victor V. Atuchin, Valery V. Preobrazhenskii

**Affiliations:** 1 Laboratory of Physical Bases of Semiconductor Heterostructures Epitaxy, Institute of Semiconductor Physics, SB RAS, Novosibirsk 630090, Russia; 2 Laboratory of Molecular Beam Epitaxy of A3B5 Compounds, Institute of Semiconductor Physics, SB RAS, Novosibirsk 630090, Russia; 3 Department of Physics, Novosibirsk State University, Novosibirsk 630090, Russia; 4 Department of Electronics, St. Petersburg Electrotechnical University “LETI”, St. Petersburg 197022, Russia; 5 Laboratory of Molecular Beam Epitaxy of Elementary Semiconductors and A3B5 Compounds, Institute of Semiconductor Physics, SB RAS, Novosibirsk 630090, Russia; 6 Laboratory of Nanodiagnostics and Nanolithography, Institute of Semiconductor Physics, SB RAS, Novosibirsk 630090, Russia; 7 Laboratory of Optical Materials and Structures, Institute of Semiconductor Physics, SB RAS, Novosibirsk 630090, Russia; 8 Research and Development Department, Kemerovo State University, Kemerovo 650000, Russia; 9 R&D Center “Advanced Electronic Technologies”, Tomsk State University, Tomsk 634034, Russia; 10 Department of Industrial Machinery Design, Novosibirsk State Technical University, Novosibirsk 630073, Russia

**Keywords:** low-temperature GaAs, molecular-beam epitaxy, III-V/Si integration, self-assembled quantum dots, dislocation filter

## Abstract

The use of low-temperature (LT) GaAs layers as dislocation filters in GaAs/Si heterostructures (HSs) was investigated in this study. The effects of intermediate LT-GaAs layers and of the post-growth and cyclic in situ annealing on the structural properties of GaAs/LT-GaAs/GaAs/Si(001) HSs were studied. It was found that the introduction of LT-GaAs layers, in combination with post-growth cyclic annealing, reduced the threading dislocation density down to 5 × 10^6^ cm^−2^, the root-mean-square roughness of the GaAs surface down to 1.1 nm, and the concentration of non-radiative recombination centers in the near-surface GaAs/Si regions down to the homoepitaxial GaAs level. Possible reasons for the improvement in the quality of near-surface GaAs layers are discussed. On the one hand, the presence of elastic deformations in the GaAs/LT-GaAs system led to dislocation line bending. On the other hand, gallium vacancies, formed in the LT-GaAs layers, diffused into the overlying GaAs layers and led to an increase in the dislocation glide rate. It was demonstrated that the GaAs/Si HSs obtained with these techniques are suitable for growing high-quality light-emitting HSs with self-assembled quantum dots.

## 1. Introduction

The formation of heterostructures (HSs) based on III-V compounds on silicon substrates makes it possible to combine the advantages of novel optoelectronic devices with the technological availability of the silicon element base. There are various advantages in the monolithic integration of highly efficient light-emitting devices based on III-V HSs into silicon circuits [1]. In particular, monolithic III-V/Si integration can ensure data transmission via an optical channel [2,3,4,5], which significantly increases data processing rates [6]. The combination of III-V and Si materials is also promising in the context of creating highly efficient tandem solar cells [7]. Thus, monolithic III-V/Si integration is one of the most important issues in modern semiconductor materials science.

The structural perfection of epitaxial III-V/Si layers is a necessary condition for successful monolithic integration of III-V-based elements into silicon circuits using planar technology. In this case, the III-V layer stock thickness should be less than 4 μm, and the III-V material formation temperature should be lower than the degradation temperature of silicon elements. The constraint for the maximum thickness of the III-V layer on Si is governed by the need to avoid the cracks caused by thermal stresses common in thick heteroepitaxial layers [8]. Therefore, the search for effective methods to improve the quality of epitaxial III-V/Si layers is significant for modern semiconductor technology.

The GaAs/Si system is the most promising among the III-V/Si heteropair systems. Despite the long history of GaAs/Si layer growth technology [4,9,10,11,12], the quality of currently available GaAs/Si HSs is far from the required level. The main problems in this research can be divided into: (i) the formation of threading dislocations (TDs) due to the mismatch between the lattice constants and the linear thermal expansion coefficients of film and substrate materials [13,14]; (ii) the formation of antiphase domains (APDs) due to errors in the order of the alternation of Ga and As atomic layers [12]; and (iii) the system’s surface morphology degradation compared to homoepitaxial GaAs/GaAs layers.

The APDs formation is induced by the presence of monatomic steps on the Si surface [15] and the development of a transition layer between GaAs and Si [16,17,18,19]. The problem caused by the presence of monatomic steps can be successfully solved by using Si substrates with a misorientation from the (001) plane by 2–6° in the [110] direction [9,20,21]. The high-temperature annealing of Si(001) substrate leads to the formation of a system of diatomic steps on its surface. Reductions in the APD density through the optimization of the conditions for the III–V compound layer nucleation on single-domain Si(001) surfaces were reported in several studies [16,17,22,23,24,25,26]. However, the search for efficient methods to reduce the TD density is still an ongoing issue. Furthermore, the issues of reducing the point defect concentration and the surface roughness in III–V/Si HSs also remain topical. The main techniques for improving the GaAs/Si HSs quality are common to GaAs/Si, other III-V/Si systems and Ge/Si heterosystems. The methods include: (i) annealing at selected growth stages [27] and post-growth heat treatments [28,29]; (ii) decreasing the temperature and growth rate at the initial stage, or the so-called two-stage growth [30,31]; and (iii) introducing dislocation filters (DFs), which involves inserting layers of stressed material or stressed superlattices that can help reduce the TD density [32,33].

Unfortunately, the separate use of any of the mentioned techniques does not provide perfect GaAs/Si layers appropriate for the creation of high-efficiency light emitters, photodetectors or other key devices based on III–V compounds. However, the combination of different techniques holds promise for the development of artificial GaAs/Si substrates of the required structural quality [34]. Early contributions [32,35] showed that the GaAs layers grown at a relatively low temperature (≤300 °C), so-called low-temperature (LT) GaAs, can be used as DFs. Moreover, our recent results [35] have indicated that the use of embedded LT-GaAs layers along with post-growth cyclic annealing may be efficient. In this relation, the present work is aimed at a detailed study of the effects of the combined use of DFs based on LT-GaAs, and the post-growth and cyclic in situ annealing on the structural perfection of GaAs/Si layers.

It is necessary to note that the approach to using LT-GaAs as a DF is fundamentally different from the two-stage growth approach, when nucleation layers grow at a low temperature. It is proposed that intermediate LT-GaAs layers should be introduced into GaAs grown at high temperature. As it is shown below, the use of such DFs, in combination with the post-growth cyclic annealing, makes it possible to reduce the TDs’ density in the structure despite the use of two-stage growth. For comparison, the microstructure and photoluminescence properties of the GaAs/Si layers grown with and without an inserting intermediate LT-GaAs layer are discussed. The efficiency of the combination of LT-GaAs based DFs and the post-growth cyclic annealing is clearly shown. The possible reasons for the efficiency of this approach are discussed. It is shown that the fabricated GaAs/Si HSs are suitable for the growth of highly efficient light-emitting HSs with the incorporated self-assembled InAs/AlAs quantum dots (SAQDs).

## 2. Epitaxial GaAs/Si HSs Growth

GaAs/Si HSs were grown by the molecular beam epitaxy (MBE) technique on an improved UHV chamber of MBE-setup “Shtat”-type (Ryazan, Russia). Crucible molecular sources were used to obtain flows of Ga and Si atoms, and a valve source with a cracking zone was used to obtain a flux of As_2_ molecules [36]. The flux densities of atoms of group III and molecules of group V were determined from the ion current of the Bayard–Alpert ionization gauge introduced during the measurement at the substrate position [37]. This technique makes it possible to determine the flux density value with the accuracy of ±2% and ±6% for atoms of group III and group V, respectively. The substrate temperature (*T*_S_) was monitored during the growth using a thermocouple attached to the heating element of the substrate heater. The thermocouple was calibrated by the transition temperatures of surface structures on the GaAs(001) film surface according to the procedure described previously [38]. The temperature determination accuracy using this technique is ±5 °C. To take into account the substrate material effect on its temperature under radiative heating conditions, the GaAs layers grown on a silicon substrate were used to calibrate the thermocouple. To suppress the APD formation, the p-type Si(001) substrates misoriented by 6° in the [110] direction (SIL’TRONIX Silicon Technologies, France) were used. The control of the structure evolution and surface morphology during the growth was carried out by reflection high-energy electron diffraction (RHEED) setup (home-made, ISP SB RAS, Novosibirsk, Russia). The base pressure in the growth chamber was 1–2 × 10^−10^ Torr.

**Figure 1:**
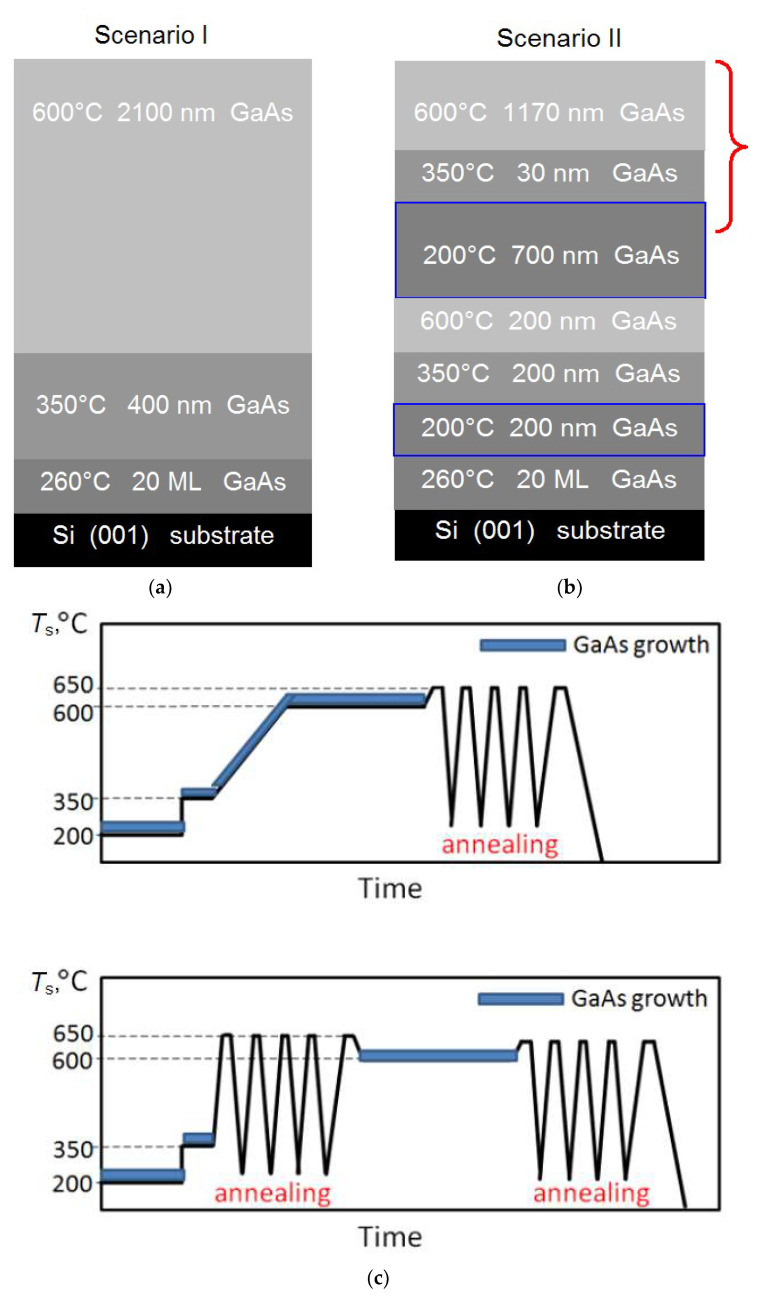
Profiles of GaAs/Si HSs grown according to Scenario I (**a**) and scenario II (**b**). The *T*_S_ values and layer thickness values are shown in the panels. The LT-GaAs layers grown at *T*_S_ = 200 °C are marked with blue lines. (**c**) The *T*_S_ sequence during the growth of the HS section marked with a red curly bracket in panel (**b**). The blue thick lines correspond to the GaAs growth points. The top panel (**c**) corresponds to the HS growth with a gradual rise in *T*_S_, and the lower panel corresponds to the HS growth with the intermediate cyclic annealing.

The oxide layer possible on the Si(001) substrate surface was removed in two stages. At the first stage, the Si surface was deoxidized at the substrate temperature (*T*_S_) of 750 °C for 20 min in an atomic Si flow corresponding to the growth rate of 0.01 monolayer per second (ML/s). At the second stage, to remove oxide residues, 5 Ga monolayers were deposited on the Si surface at *T*_S_ = 400 °C, and then the *T*_S_ value was increased to 720 °C. Under these conditions, the volatile GaO molecules are formed as a result of the chemical Ga atom interaction with SiO_x_. Next, the substrate was annealed at *T*_S_ = 850 °C for 30 min to form a system of diatomic terraces on the Si surface. After the annealing, the substrate was cooled under ultrahigh vacuum conditions to the nucleation layer formation temperature. The nucleation layer was grown by depositing 20 ML GaAs at 260 °C by atomic layer epitaxy. This method consists of the successive deposition of Ga and As atomic layers. The subsequent GaAs/Si HSs growth was carried out in two scenarios. Scenario I is shown in Figure 1a, and it includes the MBE growing the first 400 nm thick GaAs layer at *T*_S_ = 350 °C and then the second 2100 nm thick GaAs layer at *T*_S_ = 600 °C. Two annealing stages at *T*_S_ = 520 °C for 1 min with the growth stop were implemented during the first layer growth. The annealing was carried out in order to improve the epitaxial film surface morphology.

As shown in Figure 1b, the LT-GaAs layers grown at *T*_S_ = 200 °C were included in the grown HS according to Scenario II. The first LT-GaAs layer thickness is 200 nm, and the second layer is 700 nm thick. In addition, an intermediate 30 nm thick GaAs layer was grown at 350 °C just after the second LT-GaAs layer. The upper final GaAs layer thickness was as high as 1170 nm. This layer thickness was chosen from the condition that the total thickness of all HSs was ~2.5 µm. The GaAs/Si HSs fabricated according to Scenario II can be divided into two types according to the method of increasing *T*_S_ when growing the final GaAs layer. Indeed, *T*_S_ was gradually increased during the growth from 350 to 600 °C for 10 min for the HS of the first type. The cyclic annealing with the growth stopping was carried out after the formation of an intermediate 30 nm GaAs for the HS of the second type. Each cycle consisted of heating from 250 to 650 °C for 40 s, holding at 650 °C for 30 s and natural cooling to 250 °C for 2.5 min. A total of five annealing cycles was performed. The annealing procedure was performed in a flux of arsenic atoms to prevent the sample surface morphology deterioration. The arsenic flux was varied according to the current *T*_S_. The maximum heating temperature of the HSs exceeded the growth temperature by no more than 50 °C, in contrast to the high-temperature annealing discussed earlier in [28,29]. The final GaAs layer growth at *T*_S_ = 600 °C began after the last heating cycle. The cyclogram of the temperature change for each type of the HSs grown according to Scenario II is shown in Figure 1c. The postgrowth cyclic annealing, which is identical to that described above, was performed for all HSs.

The characteristics of the studied GaAs/Si HSs were compared with the characteristics of a test GaAs/GaAs structure with the thickness of 2.5 μm grown at the temperature of 580 °C. The most important features of the GaAs/Si HSs growth are listed in Table 1. The HSs grown according to Scenarios I and II without and with the post-growth annealing are designated as **I**, **I-a** and **II**, **II-a**, respectively. The HS, subjected to the additional cyclic annealing just after the second LT-GaAs layer growth, is designated as **II-a2**.

The HSs surface morphology was characterized by atomic force microscopy (AFM) using a scanning probe microscope Solver 47 (NT-MDT, Russia) in the tapping mode. The structural properties of the samples were studied by the double-crystal X-ray diffraction analysis on a DSO-1T diffractometer (Radicon Ltd., Saint Petersburg, Russia) using a Ge(004) monochromator crystal (Cu_Kα1_ emission line) and transmission electron microscopy (TEM) using a TITAN 80–300 cubed microscope (300 keV) (FEI, Hillsboro, OR, USA). The TD density was estimated from the TEM images and from the etch pits density. To form etch pits, the samples were inserted into a KOH melt at the temperature of 300 °C for 5 s. Photoluminescence (PL) was excited by a GaN laser diode with the photon energy of 3.06 eV and the power density of 25 W/cm^2^ with the use of the home-made setup (ISP, SB RAS, Novosibirsk, Russia). The steady-state PL spectra in the near-IR range were analyzed on an Acton Advanced SP2500A spectrograph (Princeton Instruments, Trenton, NJ, USA) and measured with a CCD camera cooled with liquid nitrogen. The PL measurements in the short- and mid-wavelength IR range were performed in the modulation mode using a Vertex 80 Fourier-transform infrared spectrometer (Bruker Optics, Ettlingen, Germany) equipped with an InSb photodetector cooled by liquid nitrogen. A detailed description of this measurement method is provided in [39,40]. The PL spectra in the visible and near infrared wavelength range were measured using a helium cryostat (Utreks-R, Kiev, Ukraine) in the temperature range 5–300 K with a temperature control accuracy of ±0.1 K. The PL spectra in the short- and mid-wavelength infrared range were measured using a helium cryostat (Janis CCS-150, Lake Shore Cryotronics, Westerville, OH, USA) at the temperature of 11 K.

## 3. Results

**Figure 2:**
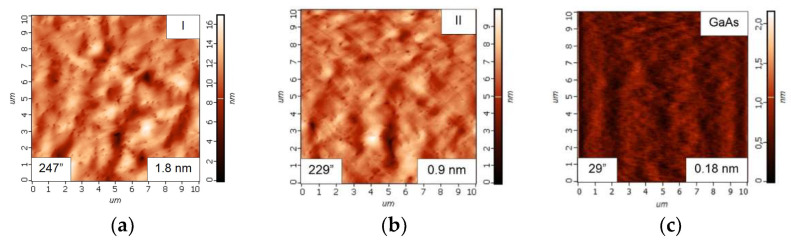
AFM images of the surface of GaAs/Si HSs **I** (**a**), **II** (**b**) and the test GaAs/GaAs structure (**c**). The root-mean-square surface roughness (RMS) and FWHM determined for the (004) peak (*W*) are shown in the bottom right and bottom left insets, respectively.

The AFM images of the surfaces of **I** and **II** HSs grown without and with the use of the LT-GaAs layer are shown in Figure 2a,b, respectively. The surface relief is isotropic for both HSs. Statistical analysis of the images sized 10 × 10 µm^2^ allows us to obtain the root-mean-square surface roughness (RMS) value. The RMS is 1.8 and 0.9 nm for **I** and **II** HSs, respectively, as indicated in the insets in the lower right corner of the panels. At the same time, the RMS value for the test GaAs/GaAs structure is only 0.18 nm, as shown in the inset of Figure 2c. The post-growth annealing does not significantly affect the GaAs/Si surface relief and only a slight increase in the RMS value up to 1.1 nm is observed for the **II-a** HS. Thus, it was shown that the use of intermediate LT-GaAs layers during the MBE growth of the GaAs/Si HSs allows the improvement of the GaAs surface relief.

The full width at a half maximum (FWHM) values were determined for the (004) peak and designated as *W.* The values are shown in the insets in the lower left corners of Figure 2a–c. The *W* values for GaAs/Si HSs **I** and **II** are 247″ and 229″, respectively, which is much higher than the *W* value found for GaAs/GaAs, which is only 29″. The *W* values for HS **I-a** and **II-a** are decreased to 196″ and 191″, respectively.

**Figure 3:**
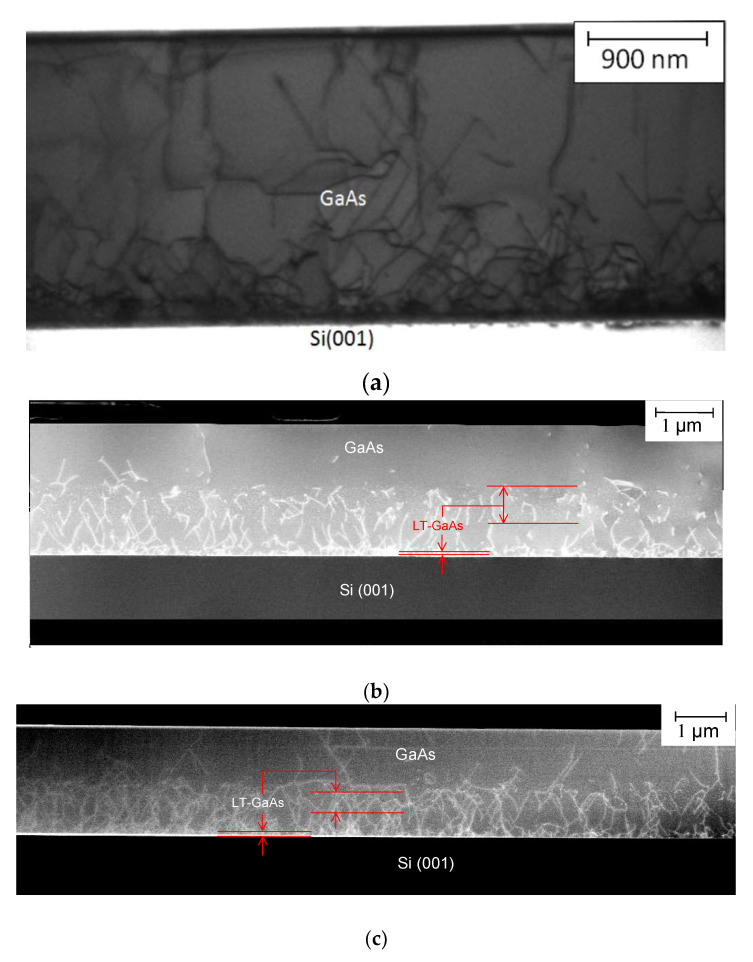
TEM images of the cross sections of **I-a** (**a**), **II-a** (**b**) and **II-a2** (**c**) GaAs/Si HSs, respectively. The LT-GaAs layer positions are indicated with red arrows.

**Figure 4:**
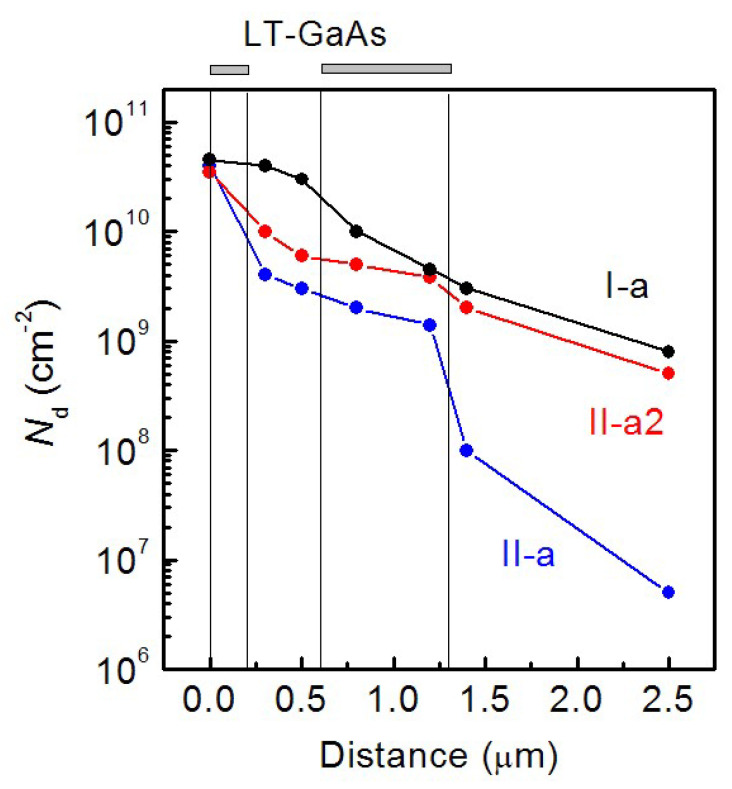
TD density dependences on the distance along the growth axis for **I-a** (black line-dots), **II-a** (blue line-dots) and **II-a2** (red line-dots) GaAs/Si HSs. The LT-GaAs layer positions are indicated with vertical lines.

The TEM images of the cross sections of the **I-a**, **II-a** and **II-a2** HSs are shown in Figure 3a–c, respectively. The statistical analysis of TEM images allows us to plot the TD density dependences over the distance along the growth axis. The dependences are shown in Figure 4. As can be seen in the figures, the LT-GaAs layer introduction has a significant effect on the TD density distribution in the HS. The TD density value near the GaAs/Si interface is more than 10^10^ cm^−2^ for all HSs. In addition, the TD density is decreased with the distance increase for all HSs. In the case of the **I-a** HS, the TD density is decreased according to a law close to the exponential one and reaches a value of about 9 × 10^8^ cm^−2^ near the GaAs surface. An abrupt TD density decrease is observed in the GaAs layers grown immediately after LT-GaAs in the HSs with LT-GaAs layers. The rate of this sharp TD density decrease exceeds the rate of a smooth TD density decrease, which is observed for the HS without the LT-GaAs layers. The TD density in the **II-a2** HS exceeds the TD density in the **II-a** HS by no more than two times at the thickness range of 0–1.3 µm. This difference is significantly enhanced by a much faster TD density decrease in the GaAs layers grown after the second LT-GaAs layer in the **II-a** HS. The TD surface density for the **II-a** HS is as low as 5 × 10^6^ cm^−2^, and for **II-a2** 5 × 10^8^ cm^−2^. The TD density in the upper GaAs layer for the **II-a** HS, determined from the etch pits density, is about 10^6^ cm^−2^, which is in a good agreement with the TEM data. The etch pits density for other HSs could not be determined because of a very high (more than 10^8^ cm^−2^) TD density in the near-surface GaAs layer. Thus, it was shown that the use of the intermediate LT-GaAs layers during the MBE growth of GaAs/Si HSs allows the reducing of the surface dislocation density. It is necessary to provide a gradual *T*_S_ increase after the second LT-GaAs layer growth for the best result.

**Figure 5:**
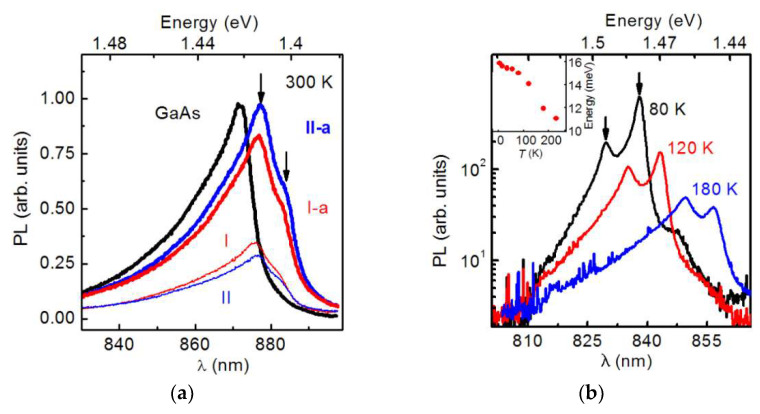
(**a**) Steady-state PL spectra of the GaAs/Si HS and the GaAs/GaAs test structure measured at 300 K. The spectra of various HSs are marked with the corresponding symbols (see Table 1). The arrows indicate the peak positions of the PL bands for GaAs/Si. (**b**) The PL spectra of the GaAs/Si **II-a** HS measured at different temperatures. The energy difference the between two PL peaks is shown in the inset as a function of temperature.

The steady-state PL spectra of the HSs were measured in a wide temperature range of 5–300 K. The spectra of HS **I**, **II**, **I-a**, **II-a** HSs and the GaAs/GaAs test structure, as measured at 300 K and at excitation power density 25 W/cm^2^ under the condition of nonresonant excitation, are shown in Figure 5a. The test GaAs/GaAs structure spectrum consists of the band with the maximum at 1.422 eV, associated with the interband electron-hole recombination [41]. A similar PL band dominates in the spectra of all GaAs/Si HSs, but it is shifted to low-energies and has a maximum at 1.413 eV. Moreover, an additional weak PL band, shifted to the low-energy region by 11 meV, appears in the GaAs/Si HS spectra. The PL peak positions in the spectra of GaAs/Si HSs are marked with arrows in Figure 5a. The **II-a** HS, grown with the use of LT-GaAs layers, shows the maximal PL intensity among all GaAs/Si HSs, and its PL intensity is comparable to that of the test GaAs/GaAs structure. The PL intensity obtained for the **I-a** HS grown without LT-GaAs layers is slightly lower and it is more than 80% of the PL intensity of the test GaAs/GaAs structure. At the same time, GaAs/Si HSs **I** and **II** fabricated without post-growth annealing are characterized by a significantly lower PL intensity, which is only 33 and 28% of the PL intensity of the test GaAs/GaAs structure. Thus, it was shown that the **II-a** HS is characterized by the best PL intensity among the GaAs/Si HSs under consideration.

The PL spectra of the **II-a** HS were measured in a wide temperature range from 11 K to 300 K in order to elucidate the nature of the low-energy shift of the PL band and the appearance of the second PL band. The measured spectra are shown in Figure 5b. As is seen, the thermal quenching of PL due to the increase in the non-radiative recombination rate occurs with the temperature increase. Over the entire temperature range, the PL band has two peaks marked with arrows in Figure 5b. An increase in temperature leads to a PL bands shift to lower energies, and it is due to the narrowing of the GaAs band gap [42]. The energy difference between the PL components is decreased with the temperature increase from 16 meV at 11 K to 11 meV at 300 K, as shown in the inset of Figure 5b. In addition, the decrease in the relative intensity of the low-energy PL peak is observed with the temperature increase.

A similar splitting of the inter-band PL band recorded for the GaAs grown on a Si substrate was observed in [43]. According to the results of this work, the splitting is attributed to the reducing of the sub-bands’ degeneracy of light and heavy holes due to the uniaxial strain of the GaAs layers. The deformations are due to the difference in the thermal expansion coefficients of GaAs and Si. We calculated the hole sub-band splitting in the framework of the model-solid approach [44]. The details of the calculations are presented in Appendix A. It was found that the splitting of hole sub-bands corresponds to a tensile strain in the HS plane and a compressive strain along the growth axis. In this configuration, the light-hole sub-band has the maximum energy in the GaAs valence band. Following the calculation results, the deformation value *ε*_xx_ in the HS plane is estimated as 1.9 × 10^−3^ at 77 K, and it varies from 2.0 × 10^−3^ at 11 K to 1.3 × 10^−3^ at 300 K. The observed increase in the relative intensity of the high-energy PL band with temperature is related to the thermal activation of holes from the light-hole sub-band to the heavy-hole sub-band.

**Figure 6:**
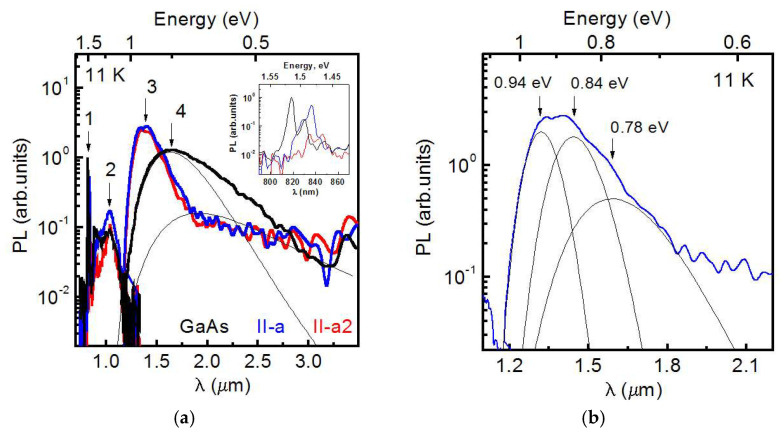
(**a**) Low-temperature (11 K) steady-state PL spectra of the GaAs/GaAs test structure (black), GaAs/Si HS **II-a** (blue) and **II-a2** (red) measured at 11K. The edge PL bands and the PL bands associated with deep level-assisted recombination are marked as 1, 2, 3 and 4, respectively. The edge PL bands at about 1.5 eV are shown in the inset. Thin black lines show the deconvolution of the PL band 4 in the GaAs/GaAs spectrum. (**b**) The deconvolution of the PL band 3 for the GaAs/Si **II-a** HS spectrum. The sub-bands with the maxima at 0.94, 0.84, and 0.78 eV are shown with thin black lines.

The low-temperature (11 K) steady-state PL spectra of the GaAs/GaAs test structure and GaAs/Si **II-a** and **II-a2** HSs, as measured in a wide wavelength range up to 3.5 μm, are shown in Figure 6a. The PL spectrum of bulk GaAs is shown by the black curve, and the GaAs/Si **II-a** and **II-a2** HS spectra are shown by the blue and red curves, respectively. The edge PL band, centered at an energy of about 1.5 eV and marked with the arrow 1 in the figure, is presented in all spectra. The PL spectra measured at energies near 1.5 eV are shown in detail in the inset of Figure 6a. The PL spectrum of the GaAs/GaAs test structure has two peaks at 1.514 and 1.493 eV, associated with the exciton recombination [45,46] and recombination of free electrons with holes localized on neutral acceptors [47], respectively. The exciton PL band for GaAs/Si HS **II-a** is split into two sub-bands at 1.497 and 1.482 eV, and it corresponds to the valence band splitting due to the residual strain in GaAs/Si, as discussed above. The low-energy band dominance is in a good agreement with the temperature dependence of the electron-hole recombination in the GaAs/Si HS, as shown in Figure 5b. The weak PL band at 1.468 eV is associated with the recombination of free electrons and neutral acceptors, as in the test GaAs/GaAs structure, but it is shifted for the spectral correction due to the residual strain. The edge PL spectrum for the GaAs/Si **II-a2** HS consists of the same split exciton bands as in the **II-a** HS, but is shifted to 1.503 and 1.486 eV for the high- and low-energy sub-bands, respectively. This weak spectral shift is attributed to a small difference in the residual strain of **II-a** and **II-a2** HSs. We also attribute the PL band at 1.465 eV to the recombination of free electrons and holes localized at neutral acceptors.

The PL bands centered at 1.2 eV are present in the PL spectra of all HSs. In Figure 6a, these bands are marked with arrow 2. According to earlier results [48,49], this band is related to the e-*V*_Ga_ transitions between the conduction band and deep levels associated with gallium vacancies. The additional PL band observed at 1.137 eV in the spectrum of the GaAs/GaAs structure is attributed to the same transitions between the conduction band and *V*_Ga_ levels, but in a different charge state *e*—*V*_Ga-_ [49,50].

At this point, the similarities between the PL spectra of the GaAs/GaAs and GaAs/Si HSs are finished and the differences should be considered. The spectrum of the GaAs/GaAs structure contains a wide PL band over the energy range of 0.5–1 eV. In Figure 6a, this band is indicated with arrow 4. This band consists of two sub-bands with the maxima at 0.65 and 0.75 eV, due to the recombination of charge carriers through the EL2 defects levels [51] and the charge carriers’ recombination through the levels of antisites in GaAs [49,52,53,54]. At the same time, the PL spectra of the GaAs/Si HS do not contain these bands, but show a PL band centered at 0.9 eV, marked with arrow 3 in Figure 6a. Taking into account the published results of studying the PL effects in the GaAs/Si layers [55], this band can be fitted by the superposition of sub-bands peaking at 0.94, 0.84 and 0.78 eV, as shown in Figure 6b. The high-energy sub-band at 0.94 eV is due to the charge carriers’ recombination through the levels of defects *V*_As-_ and *V*_Ga-_, and the sub-bands at 0.84 and 0.78 eV are associated with the charge carriers’ recombination through the levels of interstitial As (*As*_I_) and *V*_Ga_. Thus, it was shown that the GaAs/Si HSs have their specific defect atmosphere in the near-surface region in comparison with the homoepitaxial GaAs/GaAs layer.

Now, after the clarification of the PL bands, it is topical to consider the relations between their intensities. The difference of total integrated PL intensities of GaAs/GaAs and **II-a** HS is less than 3%, and it indicates the comparable non-radiative recombination level in these HSs. This is in quite a good agreement with the PL data obtained at 300 K and presented in Figure 5a. The total integrated PL intensity of II-a2 HS consists of 80% from the total integrated PL intensity of II-a HS. This difference indicates a proportionate increase in the non-radiative recombination rate in the **II-a2** HS. At the same time, the integrated intensity of the edge PL for the **II-a2** HS is more than an order of magnitude lower than that for the **II-a** HS. This is caused by an increase in the non-radiative, as well as radiative, recombination rates in the **II-a2** HS, and this effect is associated with defects.

## 4. Discussion

It is most interesting to consider the effect of LT-GaAs layers and post-growth cyclic annealing on the main GaAs/Si properties, including the TD density in the near-surface region, surface morphology and point defects concentration in the near-surface region of HSs, and that is necessary for the use of the GaAs/Si HSs for the monolithic III-V/Si integration. As was shown by the TEM observation, the introduction of LT-GaAs layers leads to a decrease in the TD density to the level of 5 × 10^6^ cm^−2^ in the GaAs layer grown on LT-GaAs for the **II-a** HS. In this case, the X-ray rocking curve width is weakly dependent on the presence of LT-GaAs layers. This is due to the fact that the X-ray diffraction method is integrating (the signal is collected from the entire GaAs/Si HS volume), and the regions with a high (10^9^–10^10^ cm^−2^) TD density make a significant contribution to the rocking curve broadening. Despite a significant decrease in the TD density, the use of LT-GaAs does not lead to significant changes in the non-radiative recombination rate in the GaAs near-surface layer. This follows from a comparison of the PL data for HSs without and with LT-GaAs layers, respectively. Taking into account the difference in the TD density in the near-surface regions of these HSs, we can conclude that point defects play the main role in the non-radiative recombination in contrast to dislocations. As the structural perfection of the GaAs/LT-GaAs film is improved, the root-mean-square roughness of its surface decreases from 1.8 nm for the type **I** HS to 0.9 nm for the type **II** HS. The improvement in the relief parameters can be partly associated with a decrease in the TD density. As is suggested, the points of dislocation emerging on the surface can create conditions for preventing the surface smoothing during the growth and post-growth annealing. Such points can, for example, contribute to the terraces echelon formation [56].

Now to discuss the effect of post-growth cyclic annealing, in combination with the use of LT-GaAs layers, on the crystalline properties of GaAs/Si HSs. The post-growth annealing leads to a decrease in the X-ray rocking curve width from ~240″ to ~190″, and it reflects an improvement in the structural perfection of the GaAs/Si HS. The PL data obtained for the samples subjected to the annealing after the growth demonstrate a decrease in the non-radiative recombination rate in the GaAs near-surface region up to the level comparable to that of homoepitaxial GaAs layers. This is in a good agreement with the data from the literature, demonstrating a decrease in the point defects concentration as a result of annealing [57]. The effect of cyclic annealing in the HS without LT-GaAs is weaker than that in the HS grown with the use of LT-GaAs. Taking into account that HSs without LT-GaAs are characterized by a higher TD density, it is possible to conclude that the non-radiative recombination in the surface regions is partly due to the presence of dislocations. The annealing procedure leads to an insignificant surface relief evolution with an increase in the RMS value from 0.9 to 1.1 nm. It should be noted that the maximal annealing temperature exceeds the GaAs growth temperature by only 50 °C. This promotes compatibility with many other manufacturing processes commonly used in III-V and Si industries.

The decrease in the TD density in epitaxial HSs with LT-GaAs can be induced by several effects. As is evident in the PL results (Figure 6), the low-temperature PL spectra of GaAs/Si HSs contain the bands associated with the presence of *As*_I_, *V*_As_ and *V*_Ga_. These bands are absent in the PL spectra of homoepitaxial GaAs. The appearance of these point defects in the near-surface regions of GaAs/Si HSs may be caused by the presence of LT-GaAs layers. Indeed, it is known that LT-GaAs layers, formed at a substrate temperature below 300 °C, contain non-stoichiometric arsenic at a level of ~1.5 at % [58,59]. A significant number of point defects are formed in the crystal during the growth, including arsenic in the site of gallium (*As*_Ga_), *As*_I_, *V*_Ga_ and complexes of these defects [58]. Commonly, the As_Ga_ defects are dominant, with a concentration of up to 10^20^ cm^−3^, and their presence explains the violation of the crystal stoichiometry and the related lattice constant increase [58,59,60,61]. The charged As_Ga+_ defects can form complexes with *V*_Ga_ [60,62]. Note that a decrease in the growth temperature from 300 to 200 °C leads to an increase in the *V*_Ga_ concentration by more than an order of magnitude (from 6 × 10^16^ to 2 × 10^18^ cm^−3^) [62]. However, the *As*_Ga+_+*V*_Ga_ complexes decompose and excess arsenic is collected in precipitates (clusters) by the annealing of LT-GaAs at 400 °C and higher temperatures [63] The free vacancies diffuse to the interfaces and other places capable of capturing them [64].

The presence of non-stoichiometric arsenic in the composition of LT-GaAs layers leads to an increase in the lattice constant of the layers to 5.659 A [58,59], which slightly exceeds the free GaAs crystal lattice constant (5.653 A [13]). This leads to the appearance of strains in the LT-GaAs/GaAs layer system. It is well known that the introduction of strained layers into heteroepitaxial III-V/Si layers leads to the bending of TD lines [65,66]. This effect opens a possibility of the TD density reduction. Indeed, as the experimental TEM data show, an abrupt decrease in the TD density occurs in the GaAs layers grown just after the LT-GaAs layers.

The presence of *V*_Ga_ in LT-GaAs layers at a concentration exceeding the equilibrium level for GaAs can also affect the TD system. We assume that *V*_Ga_, diffusing from LT-GaAs into the overlying GaAs layers during the growth and post-growth annealing, facilitate the TD glide. This, in turn, contributes to a decrease in the TD surface density. A comparison of the growth conditions applied for the II-a and II-a2 HSs, as well as the TEM patterns obtained for these HSs, is in favor of this assumption. Indeed, in the case of the II-a2 HS, after the growth of the second LT-GaAs layer and a thin 30 nm GaAs layer, the growth process was terminated and the cyclic annealing was performed. By the annealing treatment, a significant part of *V*_Ga_ was diffused to the surface and annihilated with Ga adatoms. Therefore, the subsequent GaAs layers were grown at a *V*_Ga_ concentration much lower than that in LT-GaAs. In the case of the II-a HS, the growth was not stopped, and the substrate temperature was smoothly increased during the growth of GaAs layers. The *V*_Ga_ diffusion from LT-GaAs occurred simultaneously with the GaAs layer growth, which made it possible to maintain the *V*_Ga_ concentration in the growing layer at a level above the equilibrium level. As the TEM patterns show (Figure 3 and Figure 4), the TD density in the near-surface layers of the II-a HS is almost two orders of magnitude lower than in the II-a2 HS. This indicates a noticeable increase in the TD slip rate during the epitaxial growth and cyclic post-growth annealing in GaAs layers with a higher *V*_Ga_ concentration. Thus, the experimental results show that the combined use of the insertion of dislocation filters and the cyclic post-growth annealing on the TD system in GaAs/Si HSs induces (i) the LT-GaAs deformation caused by the presence of non-stoichiometric arsenic and (ii) an increase in the *V*_Ga_ concentration in GaAs near-surface layers. Excess *V*_Ga_ is supplied by the LT-GaAs layer. It is necessary to note that the TD density in the near-surface layers of our GaAs/Si HS corresponds to the world-class results. One of the best results is reported in [34], where the authors managed to obtain TD densities in the near-surface regions of GaAs/Si layers at the level of 7 × 10^6^ cm^−2^, which is comparable to our result of 5 × 10^6^ cm^−2^.

## 5. InAs/AlAs SAQDs on the GaAs/Si HS

The obtained GaAs/Si HSs were used as artificial substrates for growing HSs with InAs/AlAs SAQDs. Generally, HSs with SAQDs are used in different fields of modern optoelectronics [67,68,69,70,71], including high-efficient light-emitter fabrication [72,73]. The growth of HSs with SAQDs was performed in a Riber Compact 21T setup (Riber, France, Paris). The GaAs/Si HSs were covered with a protective amorphous arsenic layer for the transportation between the Shtat setup, where the HSs were grown, and the Riber Compact 21T setup. A GaAs buffer layer with the total thickness of 470 nm was grown at the *T*_S_ of 600 °C after arsenic had been distilled off in a Riber Compact 21T setup. InAs SAQDs were formed between 50 nm thick AlAs layers. The first AlAs layer was grown at the *T*_S_ of 600 °C. Then, the substrate temperature was lowered to 500 °C and InAs was deposited at the amount equivalent to 2.5 ML at the deposition rate of 0.1 ML/s. The SAQD formation was controlled by RHEED. The second AlAs layer was grown at 500 °C. The HS was covered with a 20 nm GaAs layer in order to protect AlAs from an oxidation in the air. A test HS with InAs/AlAs SAQDs on a matched GaAs substrate was grown under the same conditions.

**Figure 7:**
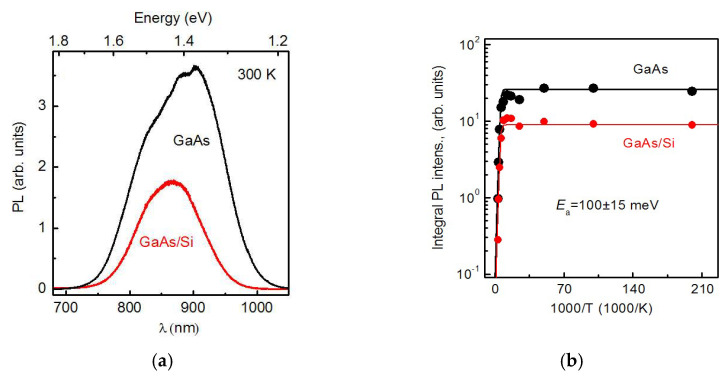
(**a**) Room temperature steady-state PL spectra of the InAs/AlAs HSs with SAQDs, as grown on the GaAs and GaAs/Si substrates. (**b**) The temperature dependences of the integral PL intensity of InAs/AlAs HSs with SAQDs grown on the GaAs and GaAs/Si substrates.

The steady-state PL spectra of the grown InAs/AlAs/GaAs/Si and InAs/AlAs/GaAs/GaAs HSs with SAQDs were measured. The PL signal was measured in the non-resonant excitation mode with a non-equilibrium charge carriers’ generation in the AlAs matrix. The PL spectra measured at room temperature are shown in Figure 7a. The PL bands with a maxima around 1.4 eV are associated with the charge carriers’ recombination in the SAQDs, and the bands are dominant in the spectra of both HSs [74]. The integrated PL intensity of SAQDs grown on GaAs/Si substrates is three times lower, in comparison with that of the HS grown on matched GaAs substrates.

The integrated PL intensity dependences on temperature for the SAQDs grown on different substrates are shown in Figure 7b. As can be seen on the curves, the integrated PL intensity in both HSs remains practically unchanged in the temperature range of 5–140 K. The thermal quenching of the SAQD PL starts at higher temperatures. In both HSs, the quenching activation energies are equal to 100 ± 15 meV. The values are comparable to the electron localization energy in InAs/AlAs SAQDs [74]. Therefore, the SAQDs PL quenching is due to the electron ejection from the SAQDs and their subsequent escape into the GaAs layers or a capture by non-radiative recombination centers in the AlAs matrix. Thus, it is demonstrated that the GaAs/Si layers discussed in the article are suitable for the use as artificial substrates in the growth of light-emitting HSs with SAQDs.

In addition, in [75], it is reported to obtain an efficient laser structure on GaAs/Si layers with a TD density of about 7 × 10^6^ cm^−2^, which is comparable to the near-surface TD density in our GaAs/Si layers. According to the results of [76], the TD density at the level of even 10^8^ cm^−2^ already allows the formation of efficient laser structures using SAQDs, the lifetime of which is about 10,000 h. Judging by the same estimates, the TD density at the level of 10^7^ cm^−2^ increases the lifetime by another three orders of magnitude, up to 10^7^ h. Thus, it can be seen that our result (5 × 10^6^ cm^−2^) fully satisfies the requirements for laser structures. Moreover, [77] reports on the creation of an InAs/InGaAs photodiode integrated on a Si substrate using GaAs/Si layers, the TD density of which is 8 × 10^6^ cm^−2^. Additionally, in [78], the creation of an InP/Si photodiode is discussed. The dislocation density in the InP layers is about 3 × 10^8^ cm^−2^, which significantly exceeds that obtained by us. Therefore, our buffer layers are also suitable for the photodiode structures fabrication.

## 6. Conclusions

In this work, the influence of the introduction of LT-GaAs layers and post-growth and cyclic in situ annealing on the crystalline properties of GaAs/Si HSs were studied. It was shown that the use of the intermediate LT-GaAs layers, along with the cyclic post-growth annealing, allows the obtaining of high-quality GaAs/Si HSs. These HSs are characterized by a TD density in the near-surface regions of 5 × 10^6^ cm^−2^, the surface RMS value of 1.1 nm, and the concentration of non-radiative recombination centers in the near-surface regions comparable with the homoepitaxial GaAs layers. The possible mechanisms, determining the efficiency of these technological methods, were discussed. First, the presence of a strain in the GaAs/LT-GaAs system contributes to the bending of the TDs along the GaAs/LT-GaAs interface. Second, the presence of *V*_Ga_, generated in LT-GaAs layers and diffused into near-surface GaAs layers, increases the TD slip rate during the epitaxial growth and cyclic post-growth annealing. It was shown that GaAs/Si HSs obtained using these technological methods are suitable for application as artificial substrates for the growth of high-performance light-emitting HSs with SAQDs.

## Figures and Tables

**Table 1:** Key parameters of the growth of GaAs/Si HSs.

HS	I	I-a	II	II-a	II-a2
Growth scenario	No LT-GaAs	No LT-GaAs	LT-GaAs	LT-GaAs	LT-GaAs
Post-growth annealing	No	Yes	No	Yes	Yes
*T_S_* increasing scenario after the second LT-GaAs layer	-	-	Gradually, during the growth	Gradually, during the growth	Cyclic annealing

**Figure A1:**
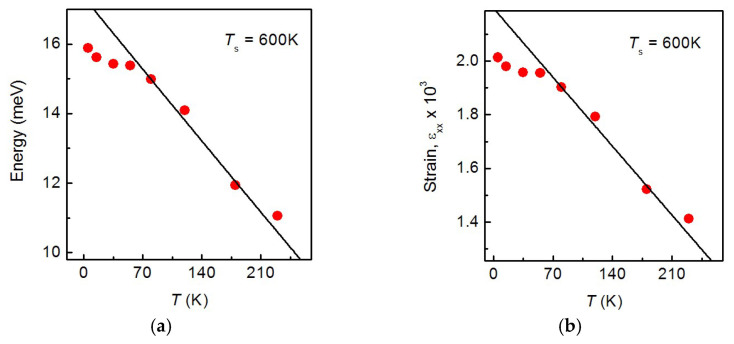
(**a**) Temperature dependence of light and heavy hole sub-bands splitting. Red dots are PL data, and the black line is calculations by formula (A13). (**b**) The calculated residual strain value of the GaAs layer as a function of temperature. The black line shows the dependence calculated by Formula (4), and the red dots show the dependence calculated using the values of the energy splitting of hole sub-bands taken from the PL data. All dependences are presented for the substrate temperature at which complete strain relaxation in GaAs occurs; *T*_S_ = 600 K.

## Data Availability

Data are available from the authors on request.

## Appendix A

The GaAs and Si lattice constants dependence on temperature is given by the expressions:

(A1) $$a_{GaAs}\left(T\right)=a_{GaAs}\left(300K\right)+\alpha_{GaAs}^{T}\cdot \left(T-300K\right)$$

(A2) $$a_{Si}\left(T\right)=a_{Si}\left(300K\right)+\alpha_{Si}^{T}\cdot \left(T-300K\right)$$

where $a_{GaAs}\left(300K\right)$ and $a_{Si}\left(300K\right)$ are the lattice constants of GaAs and Si at 300 K, $\alpha_{GaAs}^{T}$ and $\alpha_{Si}^{T}$ are the linear thermal expansion coefficients for GaAs and Si. The strain relaxation occurs due to the introduction of dislocations during the growth at *T*_S_ value. Additionally, the GaAs lattice constant varies according to *T*_S_. As the HS cools, the GaAs layer deforms according to the thermal Si substrate expansion:

(A3) $$a_{GaAs/Si}\left(T\right)=a_{GaAs}\left(T_{S}\right)+\alpha_{Si}^{T}\cdot \left(T-T_{S}\right)$$

The thermal strain in the structure plane is determined by the difference between the lattice constants of GaAs on Si and GaAs on GaAs:

(A4) $$\varepsilon_{xx}\left(T\right)=\varepsilon_{yy}\left(T\right)=\frac{a_{GaAs/Si}\left(T\right)-a_{GaAs}\left(T\right)}{a_{GaAs}\left(T\right)}=\frac{a_{GaAs}(300)+\alpha_{GaAs}^{T}\left(T_{S}-300\right)+\alpha_{Si}^{T}\left(T-T_{S}\right)}{a_{GaAs}(300)+\alpha_{GaAs}\left(T-300\right)}-1$$

The strain along the growth axis is determined from the Poisson relation [44]:

(A5) $$\varepsilon_{zz}=-2\cdot \frac{C_{12}}{C_{11}}\cdot \varepsilon_{xx}$$

where *C*_ij_ are elastic constants. All calculation is performed in the framework of model-solid approach [44].

The hydrostatic part of the strain is:

(A6) $$H=Tr(\varepsilon )=2\varepsilon_{xx}\left(1-\frac{C_{12}}{C_{11}}\right)$$

The shear strain is:

(A7) $$I=\varepsilon_{zz}-\varepsilon_{xx}=-\varepsilon_{xx}\left(2\frac{C_{12}}{C_{11}}+1\right)$$

The conduction band edge shift is:

(A8) $$\Delta E_{c}=a_{c}H$$

where $a_{c}$—hydrostatic deformation potential for the GaAs conduction band at the Г point of the Brüllien zone.

The shift of the heavy hole band top is calculated from the expression:

(A9) $$\Delta E_{hh}=a_{v}H+\frac{1}{3}\Delta_{SO}-\frac{1}{2}\delta E_{001}$$

where $a_{v}$ is the hydrostatic deformation potential for the GaAs valence band at the Г point of the Brullien zone, $\Delta_{SO}$ is the spin-orbit splitting in the GaAs valence band, and $\delta E_{001}=2bI$, where b is the shear deformation potential for the GaAs valence band.

The shift of the light holes band top can be represented as:

(A10) $$\Delta E_{lh}=a_{v}H-\frac{1}{6}\Delta_{SO}+\frac{1}{4}\delta E_{001}+\frac{1}{2}\sqrt{\Delta_{SO}^{2}+\Delta_{SO}\delta E_{001}+\frac{9}{4}\delta E_{001}^{2}}$$

The energies corresponding to two edge luminescence bands in strained GaAs/Si are given by:

(A11) $$E_{e-hh}\left(T\right)=E_{g}(T)+\Delta E_{c}\left(T\right)-\Delta E_{hh}\left(T\right)$$

(A12) $$E_{e-lh}\left(T\right)=E_{g}(T)+\Delta E_{c}\left(T\right)-\Delta E_{lh}\left(T\right)$$

The PL bands splitting energy is:

(A13) $$\Delta E_{PL}(T)=E_{e-hh}(T)-E_{e-lh}(T)=\Delta E_{lh}(T)-\Delta E_{hh}(T)$$

The material parameters used for the calculations are presented in Table A1.

**Table A1:** Material parameters used in calculations. $a_{GaAs}\left(300K\right)$ —GaAs lattice constant at 300K, $\alpha_{GaAs}^{T}$ and $\alpha_{Si}^{T}$ —linear thermal expansion coefficients of GaAs and Si, *C*_11_ and *C*_12_—GaAs elastic constants, $a_{c}$, $a_{v}$ —hydrostatic deformation potentials for the GaAs conduction band and valence band, b —shear deformation potential for the GaAs valence band, $\Delta_{SO}$ is the spin-orbit splitting in the GaAs valence band.

Parameter	Value
$a_{GaAs}\left(300K\right)$	5.65325 (Å) ^a^
$\alpha_{GaAs}^{T}$	3.88 × 10^−5^ (Å/K) ^a^
$\alpha_{Si}^{T}$	1.81 × 10^−5^ (Å/K) ^b^
*C*_11_	1221 (GPa) ^a^
*C*_12_	566 (GPa) ^a^
$a_{c}$	−7.17 (eV) ^a^
$a_{v}$	−1.16 (eV) ^a^
b	−2 (eV) ^a^
$\Delta_{SO}$	0.341 (eV) ^a^

^a^ [13], ^b^ [79].

In these calculations, there is one free parameter, namely, the substrate temperature *T*_S_, at which there was a complete strain relaxation in the GaAs/Si layers. We varied this parameter and found that the best agreement between the experimental PL data and the calculated splitting of the sub-bands of light and heavy holes is reached at *T*_S_ = 600 K (327 °C).

The calculated dependence of the splitting value on temperature is shown in Figure A1a. We attribute the discrepancy between the experimental data and calculations at low temperatures to the following factors: (i) the exciton effect, which was not taken into account in the calculations; (2) the difference in the main parameters of the GaAs material at 300 K and at helium temperatures. Alas, we know the values of these parameters only at room temperature. The dependence of the amount of strain on temperature calculated by Formula (4) (solid line) is shown in Figure A1b. Taking into account the insignificant discrepancy between the experimental PL data and the calculated values of the hole sub-bands splitting, we also calculated the strain value based on the experimental values of the splitting value (red dots in Figure A1b). As can be seen from the figure, the strain value reaches 2 × 10^−3^ at low temperatures.
